# Framing Future of Work Considerations through Climate and Built Environment Assessment of Volunteer Work Practices in the United States Equine Assisted Services

**DOI:** 10.3390/ijerph181910385

**Published:** 2021-10-02

**Authors:** Kimberly Tumlin, Sa Liu, Jae-Hong Park

**Affiliations:** 1 Department of Epidemiology, College of Public Health, University of Kentucky, Lexington, KY 40536, USA; 2 School of Health Sciences, College of Health and Human Sciences, Purdue University, West Lafayette, IN 47907, USA; saliu@purdue.edu (S.L.); park895@purdue.edu (J.-H.P.)

**Keywords:** volunteer workers, workplace design, ventilation, microenvironment, equestrian

## Abstract

The foundation of healthy workplace design is an understanding of work practices. Volunteers comprise the majority of the workforce in care centers using horses to address human health issues. Documentation is lacking on protections for worker well-being in equestrian microenvironments which are known to have the potential for dust exposures. Climate acts as a master variable in equestrian facility design and ventilation usage to address dust and temperature concerns. Using climate as an independent variable, our objective was to characterize space usage, safety, environmental control, and organizational practices through a national survey of equine assisted programs. We found that more fully enclosed indoor arena spaces were in cold/very cold and mixed-humid climates (*p* = 0.0114). Annually more volunteers (*p* = 0.0073) work in these two climate groups averaging 100 volunteers per location. A total of 34% of all facilities, regardless of climate, do not use mechanical ventilation systems (e.g., fans). As volunteer worker time in the arena increased, time in the barn microenvironment tended to decrease (*p* = 0.0538). We identified facility designs, ventilation usage, and worker arrangements to refine the scalability of future air contaminant monitoring and to provide frameworks for education, workplace design, and prevention of exposure to dust.

## 1. Introduction

In Equine Assisted Services (EAS), hippotherapy (HT) and therapeutic riding (TR) are well documented to aid in social, emotional, and behavioral development [1,2]. These programs are primarily offered at non-profit facilities, relying on volunteer workers to fill staffing gaps, and many facilities are mostly or fully volunteer-run. The Professional Association of Therapeutic Horsemanship (PATH) International reported that over 61,600 volunteers contributed an estimated value of more than four billion dollars in volunteer hours in 2017 [3]. These workers are essential in offering safe interventions for individuals with special needs and usually work in teams of three volunteers and one horse (volunteer horse unit; VHU; Figure S1). HT is a physical, occupational, or speech intervention that is delivered by a credentialed therapist and focuses on specific patient outcomes required by a physician. TR is adapted to populations that can have physical, social, or emotional vulnerabilities that the intervention is designed to mitigate [4]. Populations served by these health-based activities are predominantly youth aged 6–18 years with over 37,000 participants internationally in 2017 [3]. Organizations offering HT and TR have expanded rapidly in recent years, thus driving demand for more volunteer hours [5]. Emphasis on participant outcomes has overshadowed volunteers’ workplace health and well-being.

The United States Centers for Disease Control and Prevention (CDC) and the National Institute for Occupational Safety and Health (NIOSH) launched their Future of Work (FOW) Initiative in 2020 [6]. Overarching goals of the Initiative include promoting research concerning new job arrangements (EAS’s largely volunteer workforce serves as an obvious example) and connecting trends in workplace and workforce changes to advance worker safety, health, and well-being [6]. The CDC/NIOSH FOW Initiative has adopted a Total Worker Health^®^ (TWH) approach to advance these goals. The TWH program is a holistic model which prioritizes safety and underscores that the design of work conditions focuses on practices that can improve worker health outcomes. These frameworks are important for application in EAS to protect volunteer health, specifically around the need to “increase implementation of evidence-based programs and practices” [7]. Part of NIOSH’s TWH program includes the National Occupational Research Agenda (NORA). NORA is a program used to stimulate research and improve workplace practices. Within the FOW initiative and NORA’s research priorities, volunteer workers are included in the goal of addressing non-standard work practices and healthy work design. Work practice issues relevant to EAS include prevention and control of dust exposures and defining the built environment. Volunteer workers are recognized as important but are not regulated by standard worker programs. As such, work practices for volunteers are undefined. Workplace conditions including space usage, safety, organization, and environmental control practices need to be defined in EAS. Space usage refers to where volunteers are conducting their activities including time spent in the location, activity in the space, when activities occur, and what else is in the space including horses. Safety practices would include a description of the physical structures, maintenance of the areas, and if there are existing exposure concerns. Organization practices include a description of the EAS programs, who is served, and if there are worker training programs intended to protect health. Finally, environmental control practices are focused on how and when methods are applied to control known or suspected exposures in the environment.

A microenvironment is a location where people spend noteworthy time daily (e.g., work, volunteer, or recreational activities) and includes indoor, semi-indoor, and outdoor settings. Microenvironment exposure analysis can be used to explain exposures across spaces to further describe spatiotemporal and air contamination data [8]. In EAS, the barn and arena are two main microenvironments where people and horses move through daily. The barn is used primarily for horse housing where horses eat, rest, and are prepared for exercise (called “working the horse”). The surfaces in the barn spaces are designed for the comfort of the horses and for people to efficiently clean (e.g., manure removal and sweeping). The arena is the primary location used for horse training and activities. Arenas can consist of fully indoor spaces (roof, four walls, and openings that can be closed; called “indoor arenas”), partial indoor spaces (roof and partial side walls; called “covered arenas”), or flat and open spaces (no structure such as a roof or sidewalls; called “outdoor arenas”). The arena also differs from the barn in that the main floor surface (known as “footing”) is specially constructed of materials that maximize horse welfare [9,10]. Indoor arenas may or may not incorporate mechanical ventilation systems [11], thus air quality can vary based on the type of ventilation [12,13,14]. Because TR and HT services are often offered at low or no cost to participants, many of the TR and HT facilities do not have the resources to implement modern ventilation systems [3].

Climate drives equestrian facility design and ventilation type. In the United States, areas with colder and more inclement climates have equestrian arenas that are more enclosed with four solid walls and a roof [15]. In climates with requirements for greater ventilation to maintain comfort, arenas were more likely to be open with removable or no sidewalls. Research by McGill et al. was the first of its kind to characterize indoor arena design by climate, yet this study did not include sufficient EAS programs to address how non-profit facilities may differ or be similar to for-profit equestrian sport. Extreme weather conditions vary by climate and with more open facility designs, workers are subjected to outdoor conditions. Climate-related occupational hazards relevant to FOW in EAS programs include increased ambient temperatures, air pollution, ultraviolet radiation, and changes in the built environment [16].

Climates that have distinct seasonal changes impacted real-time area particulate concentrations in equestrian microenvironments [12,17,18]. Personal exposures to both respirable dust and respirable crystalline silica were increased when the sand-based footing was dry and external weather conditions were also dry during summer seasons [17]. Conversely, colder months resulted in higher dust production in German indoor arenas [14]. The use of mechanical ventilation improved barn microenvironments in winter seasons, although less so in summer seasons when more natural ventilation (e.g., open doors) was used [12]. The combination of water, ventilation usage, and activity in equine facilities generated a profile of exposure akin to other agricultural activities and should be considered in determining health risks. Previous studies with riding instructors, workers that stand and teach in the indoor arena, reported episodic symptoms of rhinitis and allergic irritation during work activities. Airborne dust exposure was not determined to be a cause of reported symptoms [18]. The permissible exposure limits (PELs) for respirable dust and silica are 50 mg/m^3^ and 0.05 mg/m^3^ over an 8 h time-weighted average, respectively [19]. Respirable silica concentrations measured during work tasks completed in an indoor arena ranged from 0.01 mg/m^3^ to 0.09 mg/m^3^ and concentrations exceeding the PELs were observed [17]. Respirable dust concentrations in barn/horse housing have also been reported from less than 1 mg/m^3^ to over 15 mg/m^3^, with variability impacted by external door positions, method of sampling, time of day, presence of horse feedstuffs, and activity level in the space [12,18].

Despite known workplace hazards in EAS microenvironments, the research community has paid inadequate attention to volunteers moving through these spaces. We suspected that climate was a master variable for defining characteristics of EAS programs and would follow observations of facility design and climate made by McGill et al. [15]. We hypothesized that, at EAS facilities, factors that are related to how space is structured and used (hereafter, “space usage”); factors related to human health and safety (hereafter, “safety”); factors related to organizational management (hereafter, “organization”); and factors related to the environment (hereafter, “environmental control”) are not independent of climate. Although it is reasonable that many of the factors that our survey attempted to characterize (e.g., what type of arena ventilation system is employed, if services are offered year-round) are driven by climate, for other factors, the assumption was less reasonable (e.g., what workers were responsible for maintaining arena space?). Given the paucity of data in this domain, we compared factors across climate zones to provide an understanding of volunteer workers and their potential exposure risks in EAS programs. Therefore, the purpose of this study was to: (1) establish baseline space usage, safety, organization, and environmental control practices in EAS programs using volunteer workers to aid in contextualizing future exposure studies (e.g., What happens in the spaces? What organizational or safety practices may contribute to dust exposures?) and (2) determine relationships between space usage, safety, organization, and controls used in EAS and climate designation.

## 2. Materials and Methods

### 2.1. Survey Design

We developed a questionnaire with four sections: environmental, program demographics, volunteer workers, and horse. Within each section, topics about factors that would impact air quality and potential dust exposure in offering EAS programs were developed into questions as documented in Table 1. Topics included space usage, safety, organization, and environmental control. Questions about safety and control were adapted from prior research [15]. Questions about space and organization were developed in consultation with selected PATH International Premier Accredited facilities. Selection criteria were specified before distributing the survey. Specifically, we included the surveys when the respondents were at least 18 years of age and facilities had both an indoor or covered arena and volunteer workers delivering EAS.

### 2.2. Survey Distribution

The survey was distributed over 5 months in two phases: (1) internationally through PATH International distribution lists, and (2) through personal contact with regionally targeted facilities. Respondents were instructed that their participation was voluntary; no incentive was offered for the completion of the survey. Respondents answered questions based on their knowledge of the facility, the volunteers, and how the facility functioned. Respondents were asked to identify their role at the EAS facility (Survey S1). Each response represented a unique facility. The second phase was added in the last two months of distribution because there were no respondents in the states of Illinois, Indiana, Ohio, and Kentucky. Facilities in these four states received a second targeted distribution of the survey with the intent to develop community collaborations for future exposure assessments at facilities in these locales. Respondents were permitted to skip questions except for the age and volunteer worker criterion. Incomplete surveys were included if primary selection questions of having an indoor/covered space and using volunteers were both satisfied.

### 2.3. Climate Designation

Climate designation was coded based on climate region guides [20]. The International Energy Conservation Code and American Society of Heating, Refrigerating and Air-Conditioning Engineers utilize climate designations specifically for building and ventilation guidance. There are eight zones in the United States with several states split by climate designation [20]. Alaska is the only state in the sub-arctic climate designation and was not represented in this study. Climate designations were based on heating degree days, average temperatures, and precipitation. By definition, a degree-day measurement is calculated from the mean outdoor temperature and base temperature in a building space over a 24 h period. This measurement reflects changes in the climate and is traditionally used for designing energy demands of buildings such as heating and ventilation. While actual indoor temperatures were not measured in this study, understanding the facility location in these climate designations, and how climate is a major variable in describing workplace factors provided a framework for comparing climate and work practices in EAS. Within the United States Department of Energy document, these climate designations are identifiable to the county level; thus, coding of respondent locale was grouped based on county-level climate designation [20]. Facility latitude and longitude were used to identify the county in which the facility was located. When locations were on the border between two climate designations or were the only representative in that climate designation, the location was combined with the nearest climate designation. For instance, a facility that was in northern Minnesota (very cold designation) was grouped with cold designations as it was the only very cold designation representative.

### 2.4. Data Coding and Analysis

Three types of indoor spaces were defined within the questionnaire: (1) indoor arena (2) covered arena, and (3) barn (fully enclosed horse housing area with any combination of openings and surface types). We used a median split for time variables and coded the variables into high (more than 2 h) or low categories (less than 2 h) for analyses. The seasonality of offerings was coded into “yes” for including winter and “no” for excluding the winter season. One text entry indicated a future winter offering, so that response was coded as “no” to represent current offerings. Descriptive analyses and frequencies were determined with comparisons of climate made to each of the dependent variables using the Chi-square or Fisher’s exact test, as appropriate.

## 3. Results

### 3.1. Participants

**Figure 1:**
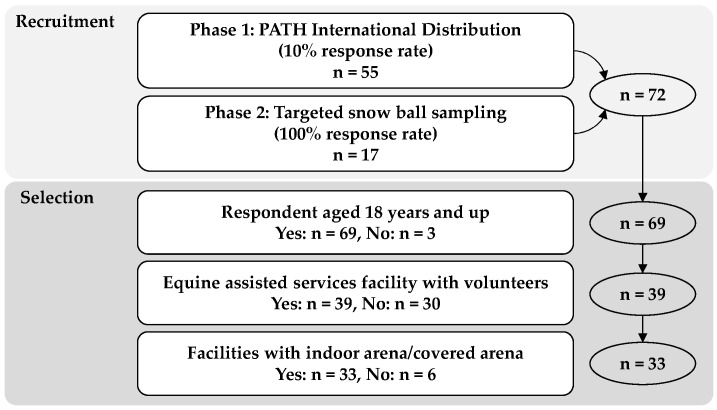
Recruitment flowchart for data analysis.

In Phase 1, 55 individual EAS facilities (10% response rate) were recruited. Following targeted recruitment, 17 more facilities responded (100% response rate). A total of 72 responses were realized (96% domestic United States). Sixty-nine facilities were represented with one respondent from each facility above 18 years. We included facilities that offered EAS and used volunteers (*n* = 39). Facilities without an indoor arena/covered arena were excluded. Within the original 72 responses, three non-domestic facilities, in Brazil, Hong Kong, and Puerto Rico were eliminated for either not having an indoor arena/covered arena, and/or for not using volunteer workers leaving a data set representing only United States responses. A total of 33 responses were used in the data analysis as shown in Figure 1.

### 3.2. Climate Designation

**Figure 2:**
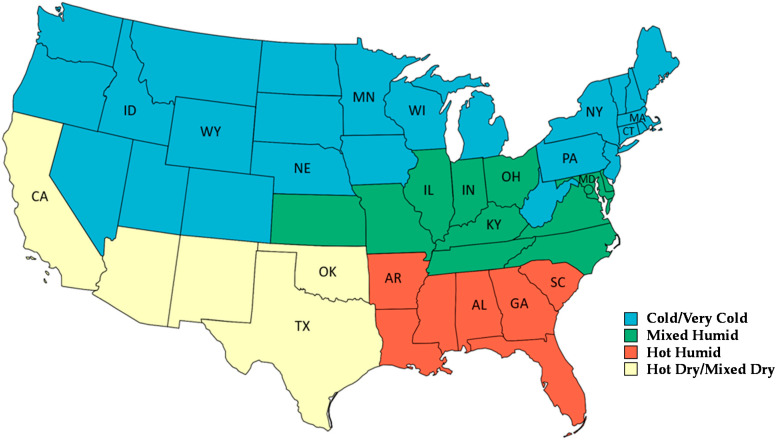
Climate designation and states in which facilities were located.

**Figure 3:**
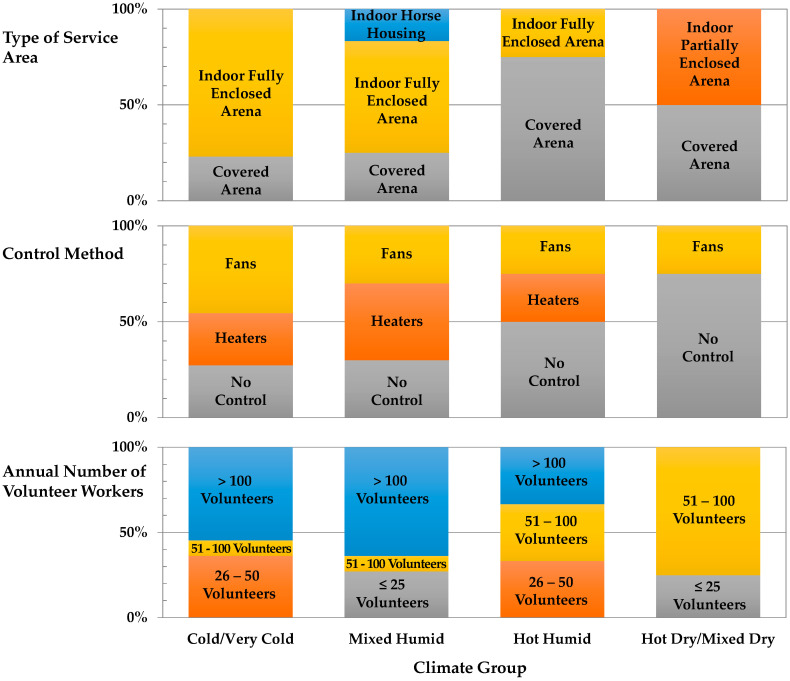
Major findings relevant to goals of the Future of Work Initiative.

Climate designations were coded based on the location latitude and longitude of the facilities. The four resulting designations were cold/very cold, mixed-humid, hot-humid, and hot-dry/mixed-dry. A total of 21 states were represented in the final data set as shown in Figure 2. Year-round services were offered at 72% (24/33) of facilities with the remaining indicating they stop activities in the winter season or rely on an assessment of weather conditions (Table 2). Facilities in hot dry/mixed dry locations offer 100% (4/4) of services year-round, whereas only 46% (6/13) of facilities in cold/very cold designations offer services year-round (*p* = 0.0250, df = 3, Χ^2^ = 9.345). A summary of major findings relevant to the overall goals of the Future of Work Initiative is depicted in Figure 3.

### 3.3. Respondent Demographics

Respondent role in the facility tended to vary by climate designation with more executive directors replying from hot dry/mixed dry and mixed humid locations. The majority of responses were completed by center staff in cold/very cold designations, with an even proportion of roles represented in hot humid designations. Volunteers were represented in both the cold/very cold and hot humid climates. The majority of respondents were female and reported as white/Caucasian as documented in Table 3.

### 3.4. Space Usage

Facilities in cold/very cold (77%; 10/13) and mixed-humid (58%; 7/12) climates reported indoor arenas that were fully enclosed (*p* = 0.0114, df = 9, Χ^2^ = 21.297). Seventy-five percent (3/4) of facilities in the hot-humid climate and 50% (2/4) in hot-dry reported using covered arenas (Table S1). Arena sizes were optionally reported, with the smallest 370.88 m^2^ (15.2 m × 24.4 m) and the largest 2613.66 m^2^ (38.1 m × 68.6 m). Facility area ranged from 4 acres (0.016 km^2^) to over 575 acres (2.33 km^2^), with an average of approximately 30 acres (0.12 km^2^).

A total of 73% (22/30) of facilities offer TR as their primary EAS activity. There was a tendency (*p* = 0.0813, df = 9, Χ^2^ = 15.366) for hot-humid locations to offer the lowest proportion of TR, but they had more private lesson programming (Table S1). To further characterize the number of people in the space over time, we analyzed questions around the numbers and frequency of volunteers moving through the spaces. There was no effect of climate on a minimal volunteer to participant ratio, with responses ranging from 1:1 to 3:1. There was a tendency for more VHU in an arena at one time in cold/very cold climates (more than 4 = 80%; *p* = 0.0775, df = 6, Χ^2^ = 11.373). The fewest average VHUs were observed in hot-humid climate facilities (less than 2 at a time = 33%). There was no interaction of how long volunteers work daily by climate designation. A relationship between the number of volunteers and climate designation was observed with cold/very cold (55%; 6/11) and mixed-humid (64%; 7/11) locations reporting more than 100 volunteers annually (*p* = 0.0073, df = 9, Χ^2^ = 22.552). To characterize work time, respondents indicated daily and weekly work hours of volunteers. Daily, there was no relationship of time spent in the indoor arena by climate; volunteer workers are in the arena for up to 5 h/day in all climate designations (“High” group). Similarly, there was no relationship of daily time spent in the barn microenvironment by climate designation. Weekly there was no relationship between weekly hours and climate in the indoor arena. There was a relationship of volunteer work in the barn space weekly (*p* = 0.0311, df = 3, Χ^2^ = 28.870). Volunteers in the mixed-humid climate more likely worked for greater than 2 h/wk in the barn space. When evaluating the relationship between volunteer worker time in the two spaces, when time in the arena is high, barn time tends to be reduced (*p* = 0.0538).

### 3.5. Safety

Concerns about the indoor environment in both arena and barn spaces were shared in nearly three-quarters of EAS facilities, and there were no differences based on climate designation. Facilities in mixed humid, hot humid, and hot dry/mixed dry shared multiple concerns over temperature, dust, humidity/moisture, and ventilation challenges. Dust was a concern in 43% (3/7) of cold/very cold and 50% (2/4) of hot humid locations (Table S2). Airborne dust sources include the footing (soil surface), proximity to vehicles, and cooking, tobacco smoking/vaping, or other aerosol liberation activities. There was no relationship by climate designation of footing (soil surface) selection (Table S2). The majority of facilities had sand as the primary base (63%; 19/30) with mixed types (including fiber, rubber, wood chip) being secondary to sand bases (30%; 9/30). Paid staff maintain the arena footing (e.g., applying water, grooming the surface, and removing manure) in 90% (26/29) of facilities. Vehicle proximity was limited in 60% (15/25) of facilities, preventing exhaust fumes from entering the arena microenvironment. Nearby parking was restricted to individuals having mobility challenges, and facilities indicated that vehicles were not left running. There is no smoking/vaping permitted in any of the indoor microenvironments (arena or barn) in 100% (27/27) of the facilities.

### 3.6. Organization

There were no differences in affiliation with professional associations by climate designation, although the majority of facilities were associated with PATH International. Collectively, additional affiliations included the Certified Horsemanship Association (10%; 3/33), American Hippotherapy Association (3%; 1/33), National Council for Therapeutic Recreation Certification (3%; 1/33), and United States Equestrian Federation (3%; 1/33). The majority (73%; 24/33) of services are provided to children under 18 years and there were no differences by climate designation in who participates in the EAS (Table S3). Respondents answered questions about their personal awareness of respiratory conditions in volunteers such as allergies, asthma, or bronchitis. Forty-six percent (13/29) responded yes, they had awareness; however, there were no relationships to climate designation by type of condition. Thirty-three percent (1/3) of facilities in hot humid locations indicated they were aware of volunteers having a horse allergy (Table S3). Proportionately, more responses in hot-dry climates were found in the “I am not aware of any health conditions of volunteers” than other climate designations. Only facilities in the mixed humid climate provide education on severe respiratory conditions and treatment (e.g., asthma, and use of respirators), whereas more than 50% (13/25) in cold-very/cold, mixed humid, and hot humid climates provide volunteer training on mild respiratory condition recognition such as seasonal allergies and general irritation (Table S3). Respondents also considered their knowledge of horse respiratory health in these spaces. There were no differences by climate designation, although most respondents indicated “I am not aware of any health conditions of horses”. Of those who indicated an answer of “No diagnosed condition, but horses will cough” respondents indicated coughing occurs in the arena 100% of the time in mixed humid, hot humid, and hot dry/mixed dry locations (*n* = 5). Cold/very cold responses were split evenly between the barn and arena microenvironment, although there were no differences by climate designation and season when horses cough (Table S3).

### 3.7. Environmental Control

Control topics were assessed through questions on arena ventilation including use of fans or heaters, number and type of fans, and when fans were used for controlling air movement. A total of 34% (10/29) of facilities do not use any type of ventilation system. When considering control methods by climate designation, there were no differences in the usage of fans or heaters. No heaters were reported in hot dry/mixed dry facilities. On average only 38% (11/29) of facilities used fans. Of those locations that used fans, box fans were commonly used in hot dry/mixed dry locations and ceiling mounted low speed, high volume fans were reported in cold/very cold and hot humid locations. In the mixed-humid facilities, both exhaust and combinations of the other fan types (box/high speed) were utilized. There were no differences by climate in the number of fans used, although cold/very cold locations reported more variability in the number of fans than other climates (Table S4). Facilities chose ventilation methods based primarily on environmental temperature and humidity (Survey S1). There were no differences by climate in when fans were used, although mixed humid locations were the only locations to respond that fans were used year-round. We evaluated time in the arena microenvironment and the relationship to the use of ventilation systems. In hot humid locations, volunteers worked more than 2 h in arena microenvironments that used fans as the primary control method (*p* = 0.0507, df = 1, X^2^ = 3.819).

## 4. Discussion

The purpose of this study was to provide baseline characterization of factors that impact volunteer worker health linked to FOW initiative priorities. To achieve this goal we utilized a survey addressing factors in EAS programs and evaluated climate as a master variable to ascertain differences among factors. Based on the analyses, there were effects of climate for many, but not all factors characterized, which supported our hypothesis. For instance, more volunteers work annually in cold/very cold and mixed humid climates than other areas. Conversely, we identified that ventilation control was independent of climate.

There is a paucity of research in EAS which focuses on defining protocols and practices at the organizational level [21]. Participant outcomes that are improved with EAS included somatic awareness, relational experiences, and brain functionality improvement [22,23,24,25]. Although, therapeutic interventions vary widely in approach (e.g., mounted or unmounted), delivery (frequency and duration), and measurement (e.g., biometric or psychometric scales). Prior EAS research does not offer a consensus on how to offer services and does not address climate dimensions addressed in our study. As such, this study is the first to establish how volunteer workers interact in equestrian spaces, and how EAS programs offer services related to climate.

Demographics of the respondent roles varied by climate. We expected more executive director responses across all climate designations, however, volunteer respondents were represented equally in the hot-humid climate designation. EAS facilities are primarily non-profit organizations and rely on volunteers to function. In this study, EAS roles were represented at the executive director, staff, and volunteer levels, suggesting that volunteers may have work roles that cross traditional expectations of offering EAS activities. Volunteers who responded to the survey were not asked what they did daily in the EAS organization.

We validated that climate drives facility design and space usage as previously reported in non-EAS equestrian facilities [15]. Facilities in cold/very cold and mixed humid climate designations operated in more enclosed structures representing indoor environments. We found that facilities in hot-dry and hot-humid climates reported more covered spaces that do not have walls; hence these are not true indoor environments. Given these findings, design and modeling of future exposure studies and worker protections should consider facility design and ventilation usage. In more open settings, dust exposure is dependent on natural airflow and arena footing moisture content is different based on footing type, ambient temperature, and moisture [9]. In a prior study, exposure to respirable dust and respirable crystalline silica was increased when conditions external to the arena were dry and no water was applied to the footing surface [17]. Changes in ambient temperature were positively correlated with variability in dust concentrations in barns [26].

Space usage also tended to be higher in cold/very cold climates at a single time point. Prior research indicates that increased activity in indoor arenas is associated with increased dispersion of footing material and subsequent increase in airborne contaminants [9,14]. In the equestrian microenvironment, air contaminants are generally produced by the activities of horses and people. The generation of air contaminants can be affected by occupancy (number of people and horses) [27,28], building characteristics (infiltration, airtightness, and ventilation system) [29,30,31], and activity type (sweeping, feeding horses, and use of bedding materials) [17]. We found that there was an inverse relationship between volunteer worker time in the arena and barn microenvironments. This finding is important to understand what activity type volunteers engage in during the offering of EAS programs and provides a baseline for determining the next steps in understanding potential exposure risks.

Despite standards of practice provided for PATH, International [32], volunteer workers’ health and safety are not addressed. EAS services are most like self-regulated organizations, where standards and practices are adapted for the delivery of services but worker health is not overseen by external bodies. Oftentimes these self-regulated organizations have complex social and political factors [33]. Because volunteers in EAS do not have the protections normally provided to standard workers, developing more detailed questioning around the adoption of health and safety practices is necessitated. Organizationally, programs hold permanent workers to standards different from temporary or casual workers, thus perpetuating organizational discrimination in health and safety practices between groups of workers [33]. To characterize potential exposure differences between standard workers and volunteers, we established that volunteer workers in EAS do not follow traditional work schedules. Time-weighted averages for exposures in volunteer workers would be based on a 5 h day/20 h workweek. This is different from the standard 8 h day used for traditional workers. 

Respondents consistently reported safety concerns that included temperature, moisture/humidity, dust, and airflow. We expected this finding to vary by climate, as the moisture content of arena surfaces affects air contaminants, with wet footing materials having lower airborne dust liberation [10]. Conversely, higher moisture combined with lower ventilation, as measured by air exchange rates, previously resulted in greater mold and bioaerosol contaminates in equestrian microenvironments [13,14]. We also characterized EAS program footing as primarily sand and that footing selection was independent of climate was novel. Prior work suggested that footing surfaces vary by climate [15]. We found that climate impacted the number of volunteers working annually in the indoor arena with the highest number of volunteers in both the cold/very cold and mixed humid climates. What differentiates mixed humid climates from cold/very cold climates is the level of precipitation. Annually mixed humid climates in the United States receive more than 50 cm (20 inches) of precipitation [20]. While not measured in this study the impact of moisture, surface materials, and air exchange rates combined with the number of volunteer workers in various climates requires definition as potential exposure risks for EAS organizations.

Integrating organizational practices which expand approaches to promote health and safety and consider the relationships between workplace and health is core to the FOW Initiative priorities. Organizational practices that support relevant worker protections such as personal protection equipment or the use of environmental controls in the physical work environment are important organizational design factors [6]. Furthermore, climate related hazards such as increased ambient temperature or exposure to air pollution require organizations to enhance risk communications [16]. At the organization level, we expected more evidence of impact on human and horse health based on climate. Interestingly, 44% (12/27) of facilities indicated they were not aware of respiratory conditions. It was also surprising to find horse allergies in a population that works with horses. This is an important finding when considering future studies on lung function. Horse allergen dispersion is related to ventilation patterns in barn microenvironments [34], so further characterization of how and when ventilation is used is needed to better understand the dispersion of allergens in the arena microenvironment. Training on respiratory conditions was independent of climate for mild respiratory conditions such as general respiratory irritation or seasonal allergies. Respondents did not indicate if EAS programs provided training on ventilation usage and recognizing when airborne dust could pose a respiratory risk. Only those facilities in mixed-humid climates reported providing training on severe respiratory conditions including the use of artificial respirators. In horse farm workers, those who never or infrequently used respirators as personal protection equipment were two times more likely to report respiratory symptoms when in barn microenvironments [35]. This survey did not specifically ask if volunteers used respirators.

Environmental control in the form of mechanical ventilation serves as a primary means to control indoor contaminants, where indoor air is exhausted and clean outside air is brought into the facility. Surprisingly, 34% (10/29) of facilities reported no specific ventilation control and of these responses. Fifty percent were located in the cold/very cold climate. Lack of specific ventilation control in equestrian indoor arenas is not uncommon [13,17], although we expected that facilities in cold/very cold climates would use have more environmental control methods (fans, heaters) than other climates.

Because organizations often rely on volunteer workers to ensure that activities in school and health programs are successful [36,37], findings from this study provide frameworks for FOW considerations of the built environment such as engineering design and ventilation recommendations, as well as addressing factors influencing worker health safety at the organizational leadership level. We successfully engaged facilities from various climate locations and demonstrated a variety of facility structures and ventilation systems. Potential dust exposure risks in indoor arenas include footing surfaces, the interplay between temperature, moisture, and airflow, and who is in the environment (e.g., horses and humans). We validated that most volunteers focused on activities including EAS participants as evidenced by the questions on VHU ratio and time spent in specific spaces. Volunteers did not work on the maintenance of the arena footing. Much of the prior exposure research has focused on activities unrelated to riding or interacting with horses such as using a tractor to drag and water the arena, general facility maintenance, or considered only a single rider in the indoor space [12,14,18]. We did not ask if other maintenance activities such as sweeping, raking, or blowing the spaces were completed by volunteers.

We also considered that seasonality would have a role in air contaminant risks. In winter seasons when barns inlets (e.g., doors, windows, ridge vents) were closed, the use of fans did not decrease respirable dust concentrations [12,13]. In climates experiencing below freezing temperatures, it is impractical to open all external air inlets as temperature control becomes a primary concern. Decreased ventilation use in winter seasons has been associated with human respiratory symptoms [12,17,36]. In our survey, we specifically asked about when fans were used for ventilation and responses of “when horses and humans are hot” and multiple reasons (temperature, dust, flies) predominated. Only those facilities in mixed-humid reported using fans year-round. In cold locations, facilities are generally enclosed with minimal ventilation since exhausted air must be replaced with cold outside air that must be heated, resulting in increased heating costs. Considering there are fewer air exchanges in cold climates to maintain internal temperatures [20], contaminants may accumulate when ventilation rates are low as previously documented [38]. Our survey indicated that EAS programs are offered year-round. Because the ambient temperature and humidity impact equestrian microenvironment air quality [10,18,34], the use of fans needs to be considered along with activity and season of activity offering. In mixed-humid climates and cold/very cold climate facilities, there are over 100 volunteers annually interacting in these spaces. EAS facilities in these two climate designations provide a natural experiment for future exposure studies to evaluate exposures in volunteer workers.

This study has several limitations. The sample size was small and no efforts were made to ensure the pool of respondents was representative of facilities that were affiliated with organizations other than PATH International, besides the regionally sampled facilities. Although demographics of respondents were recorded, demographics of volunteers such as age, sex, race, and ethnicity were not identified. These demographics would aid in further understanding exposure risks to dust exposures. Further understanding of sex and age differences in this non-standard workforce would also contribute to advancing the FOW Initiative priorities. Aging volunteers face challenges of increased risk not only for exposure but musculoskeletal injury [16] especially given differences in surfaces identified in this study. Given the complete absence of literature in this domain, however, we judge our study to be an important proof-of-concept. Our systematic characterization of the volunteer work practices, facility characterization, and relationships to climate inform the next steps for conducting exposure assessments.

## 5. Conclusions

In this study, we explored relationships of climate and volunteer worker arrangements in indoor equestrian microenvironments. The FOW Initiative highlights the importance of organizations to provide hazard-free work environments while acknowledging that exactly how these safe environments will be deployed is unclear. The FOW Initiative also designates volunteers as non-standard workers [6] who may be vulnerable to unfair treatment. Volunteers have comprised an important workforce in HT and TR programs. We have defined volunteer work practices that can be relevant to TWH prevention and control guidelines. These results can also be applied to program assessments to make the case for advancements in ventilation and engineering strategies for these non-profit programs to promote healthy workspaces. In climates with more moisture, additional contaminates beyond particulate matter could be potential exposure sources for volunteer workers. We could expect that workers in cold locations might be more exposed to indoor pollutants than workers in hot locations. To evaluate this hypothesis, further study to monitor indoor air contaminants needs to be conducted. The relationships between systems of the built environment, climate, and volunteer worker health aids in understanding potential exposure risks for this unique population. EAS programs have the opportunity to utilize these findings to address volunteer worker safety to ensure the health and well-being of all populations involved in the therapeutic process.

## Figures and Tables

**Table 1:** Selected survey topics, questions, and potential answers for each section.

**Section 1: Environmental**		
**Topic**	**Question**	**Potential Answers**
Safety	Do you have any concerns about the environment within the indoor arena/activities space?	Yes No
	Do your concerns fall into any of these categories?	Multiple (air movement, moisture, dust, temperature) Temperature Dust
	What is the main component of the surface footing in the indoor arena/activity area?	Dirt/Clay Sand/washed sand Mixed (Wood Chip/Sawdust Textile/fiber) Rubber
	What other materials are in the footing mixture?	Dirt/Clay Sand/washed sand Crushed Rock/Wood Chip Textile/fiber Rubber Waxed Sand Dust suppression additive (Magnesium Chloride)
	Who is responsible for maintaining the arena space?	Volunteers Staff Other (contracted labor; property owners/leased property)
	Is smoking allowed in the indoor arena/activity space?	Yes No
	Please characterize how frequently vehicles are near or in the barn/horse housing area.	Only farm traffic is nearby Access is limited but available to service providers (vets/farriers/feed delivery) Driving and parking next to the area is common practice
Control	Are there temperature/ventilation control methods in the indoor arena?	Fans Heaters Fans and Heaters None
	What type of fans are used?	Box fan Low speed, high velocity (ceiling mounted) Exhaust
	How many fans?	One to three Four to six Six to nine
	When are fans used for ventilation?	Year-round When horses and humans are hot Based on thermometer measurements (set heat limits) When it feels humid in the facility During fly season Multiple (temperature, dust, flies)
**Section 2: Program Demographics**		
**Topic**	**Question**	**Potential Answers**
Space Usage	Select the activity for which you utilize your facility space the most.	Adaptive Riding/Therapeutic Riding Hippotherapy Recreational Riding Competition/Boarding
	Which of the following best describes where you conduct the majority of your services?	Covered arena Indoor/partially enclosed arena Indoor/fully enclosed arena Indoor/horse housing (stalls/barn)
	How are services offered year-round at this center?	All seasons All seasons except Winter Other (text entry)
Organization	Which of the following describes your Member Center affiliation?	PATH International/Premier Center Member United States Equestrian Federation American Hippotherapy Association National Council for Therapeutic Recreation Certification Multiple Affiliations (including PATH)
	Which of the following best describes the majority of your participants who engage in your equine assisted activities or equine assisted therapies?	Children under 9 years Children under 18 years Adults above 18 years Other (text entry)
**Section 3: Volunteer Workers**		
**Topic**	**Question**	**Potential Answers**
Space Usage	What is the minimal volunteer to participant ratio for the indoor arena/activities space?	1 volunteer to 1 participant 2 volunteers to 1 participant 3 volunteers to 1 participant
	Approximately how many volunteers participate in a typical day at this center?	Less than 10 11–25 26–45
	Annually, how many volunteers participate at this center?	Less than 25 26–50 51–100 more than 100
	If applicable, how long is the volunteer in the indoor arena/activity area on a typical day at this center?	Open ended entry, recoded to: Low (<2 h daily) High (>2 h daily)
	If applicable, how long is a typical volunteer in a barn/horse housing area on a typical day at this center?	Open ended entry recoded to: Low (<2 h daily) High (>2 h daily)
	If applicable, how many hours per week does each volunteer engage in activities offered in the indoor arena/activities space?	Open ended entry recoded to: Low (<2 h weekly) High (>2 h weekly)
Organization	Are you aware if the center volunteers have any of the following respiratory conditions?	Seasonal allergy (pollen primarily) Horse allergy (dander primarily) Grass/hay allergy I am not aware of any health conditions of volunteers
	Do volunteers receive training/education about the following respiratory conditions?	Severe conditions (e.g., Asthma, use of artificial respirators) Mild conditions (e.g., Seasonal, horse, grass/hay allergy) Multiple conditions (Severe and allergy) I am not aware of any volunteer training/education in respiratory conditions
**Section 4: Horse**		
**Topic**	**Question**	**Potential Answers**
Space Usage	How many horses or horse-volunteer-participant units are in the arena space at one time?	Less than 2 on average Two to four on average More than four on average
Organization	Are you aware if the center horses involved in activities have any of the following respiratory conditions?	ROA (recurrent airway obstruction) IAD (non-infectious inflammatory airway disease) Seasonal allergy No diagnosed condition, but horses will cough I am not aware of any health conditions of horses
	If horses do not have diagnosed conditions, but will cough, then which of the following do you observe to cause the most coughing in the horses?	When in the stall/barn area During activities in the arena
	If horse coughing is observed in the stall/barn or during activities/interventions in the arena space, which time of year do you notice the majority of the coughing in the horses?	Winter Spring Summer Fall Year round

**Table 2:** Seasonality of Equine Assisted Services offered by climate designation.

	Cold/Very Cold *n* = 13	Mixed Humid * n = 12	Hot Humid *n* = 4	Hot Dry/Mixed Dry * n = 4
Year round	46%	92%	67%	100%
Spring, Summer, Fall only	46%	8%	33%	0%
Other (weather dependent)	8%	0%	0%	0%

* An effect of seasonality by climate designation was realized at *p* = 0.0250, df = 3, Χ^2^ = 9.345.

**Table 3:** Respondent demographics by climate designation.

	Cold/Very Cold *n* = 11	Mixed Humid *n* = 9	Hot Humid *n* = 3	Hot Dry/Mixed Dry *n* = 4
What is your role at this center?				
Center/Executive Director	18%	56%	33%	75%
Center Staff	63%	44%	34%	25%
Volunteer	18%	0%	33%	0%
(*p* = 0.0907, df = 9, Χ^2^ = 15.007)				
What is your sex?				
Male	0%	0%	0%	25%
Female	91%	100%	100%	75%
Prefer not to answer	0%	0%	0%	0%
(*p* = 0.4460, df = 6, Χ^2^ = 5.800)				
What is your race?				
White/Caucasian	91%	89%	67%	75%
American Indian/Alaska Native	0%	0%	0%	25%
Prefer not to answer	9%	11%	33%	0%
(*p* = 0.4400, df = 6, Χ^2^ = 5.852)				

## Data Availability

Data are contained within the article and the survey provided in the supplements.

## Supplementary Materials

The following are available online at https://www.mdpi.com/article/10.3390/ijerph181910385/s1, Figure S1: Example of a volunteer-horse-unit in a fully enclosed arena (used with permission from Healing Strides of VA, Boone’s Mill, VA, USA). Survey S1: Questionnaire utilized. Tables S1–S4 Detailed questionnaire results
