# Supplementary material for: Framing Future of Work Considerations through Climate and Built Environment Assessment of Volunteer Work Practices in the United States Equine Assisted Services
Source: Int J Environ Res Public Health. 2021 Oct 2;18(19):10385. doi: 10.3390/ijerph181910385 (PMC8508050; doi:10.3390/ijerph181910385)

Figure S1. Example of a volunteer-horse-unit a fully enclosed arena (used with permission from Healing Strides of VA, Boone's Mill, VA).

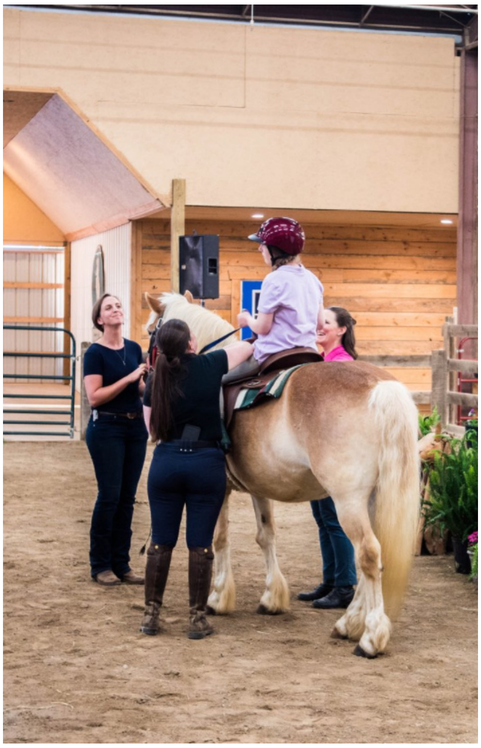

Supplement: Supplementary file 1 [file ijerph-18-10385-s001.zip › Figure S1.pdf]
