# Supplementary material for: Framing Future of Work Considerations through Climate and Built Environment Assessment of Volunteer Work Practices in the United States Equine Assisted Services
Source: Int J Environ Res Public Health. 2021 Oct 2;18(19):10385. doi: 10.3390/ijerph181910385 (PMC8508050; doi:10.3390/ijerph181910385)
Supplement: Supplementary file 1 [file ijerph-18-10385-s001.zip › Survey S1 CLEAN.pdf]

Survey S1: Copy of the full survey questionnaire utilized in this project.

Are you at least 18 years of age?

o Yes

o No

Skip To: End of Survey If Are you at least 18 years of age? = No

Where is the center located?

o United States

o Country other than United States (please list)
________________________________________________

Display This Question:

If Where is the center located? = United States

In which state is the center located?

▼ Alabama ... Wyoming

Do you utilize volunteers to offer services?

o Yes

o No

Skip To: End of Survey If Do you utilize volunteers to offer services. = No

Do you have a covered, partially enclosed, fully enclosed arena, and/or indoor horse housing space in which you offer services?

o Yes

o No

Skip To: End of Survey If Do you have a covered, partially enclosed, fully enclosed arena, and/or indoor horse housing spac... = No

## *Environmental Factors*

Please briefly describe the center environment that includes land size (acreage) and number of buildings/structures.

(Text Entry)________________________________________________________________

Do you have any concerns about the environment within the indoor arena/activities space? (ie is it dusty? Is it hot? Is there little air movement? etc)

o Yes

o No

Display This Question:

Do you have any concerns about the environment within the indoor arena/activities space? (is =Yes)

Do your concerns fall into any of these categories? (select all that apply)

▢ Lack of air movement

▢ Too much air movement

▢ Temperature

▢ Dust

▢ Moisture control

Are there temperature/ventilation control methods in the indoor arena? (Select all that apply)

▢ Heaters

▢ Fans

▢ Central air system

▢ None

Display This Question:

If Are there temperature/ventilation control methods in the indoor arena? (Select all that apply) = Fans

How many fans are there?

o 1-3

o 4-6

o 6-9

Display This Question:

If Are there temperature/ventilation control methods in the indoor arena? (Select all that apply) = Fans

What type of fans are used?

o Box fan

o Low speed high velocity (ceiling mounted)

o Drum/floor fan

o Exhaust

o Other (please specify) ________________________________________________

Display This Question:

If Are there temperature/ventilation control methods in the indoor arena? (Select all that apply) = Fans

When are fans used for ventilation of your facility? (select all that apply)

▢ Year-round

▢ When horses and humans are hot

▢ Based on thermometer measurements (set heat limits)

▢ When it feels humid in the facility

▢ During fly season

▢ When it smells/odors are present

▢ To move visible dust

▢ Other (please specify) ________________________________________________

What is the main component of the surface footing in the indoor arena/activity area? (Select the one that is predominant in the surface footing)

o Dirt/Clay

o Sand/washed sand

o Crushed Rock

o Wood Chip/Sawdust

o Textile/fiber

o Rubber

o Other

What other materials are in the surface footing mixture? (Select all that apply)

☐ Dirt/Clay

☐ Sand/washed sand

☐ Crushed Rock

☐ Wood Chip/Sawdust

☐ Textile/fiber

☐ Rubber

☐ Waxed Sand

☐ Dust suppression (Magnesium Chloride/Calcium Chloride)

☐ Other (Please specify) __________________________________________________

Who is responsible for maintaining the arena space (footing/ventilation)?

o Volunteers

o Staff

o Other (Please specify) __________________________________________________

Is smoking allowed in the indoor arena/activity space?

o Yes

o No

Please characterize how frequently vehicles are near or in the barn/horse housing area.

o Only farm traffic is nearby

o Access is limited but available to service providers (vets/farriers/feed delivery)

o Driving and parking next to the area is common practice

o Other (please specify) ________________________________________________

## *Program Demographics*

Select the activity for which you utilize your facility space the most.

o Adaptive Riding/Therapeutic Riding

o Hippotherapy

o Para-equestrian activities

o Camps (any type)

o Recreational Riding

o Non-mounted activities

o Other ________________________________________________

Which of the following best describes where you conduct the majority of your services?

o Covered arena

o Indoor/partially enclosed arena

o Indoor/fully enclosed arena

o Indoor/horse housing (stalls/barn)

Which of the following  describes your Member Center affiliation? (select all that apply)

▢ PATH International Center Member

▢ PATH International Premier Accredited Center Member

▢ American Hippotherapy Association

▢ National Council for Therapeutic Recreation Certification

▢ US Para-Equestrian/ USEF

▢ Association of Chartered Physiotherapists in Therapeutic Riding (UK)

Other ______________________________________________

Are services offered year-round at this center?

o Definitely yes (all seasons)

o Only in Spring, Summer, and Fall

o Only in Spring and Summer

o Only in Spring and Fall

o Only in Summer and Fall

o Follows school/academic year (late summer through to late spring)

Other ______________________________________________

Which of the following best describes the majority of your participants who engage in your equine assisted activities or equine assisted therapies? (select the most predominant population)

o Children under 9 years

o Children under 18 years

o Adults aged 18-30 years

o Adults aged 31-100 years

Other ______________________________________________

Are you aware if the center participants receiving instruction or specific interventions have any of the following respiratory conditions? (select all that apply)

▢ COPD

▢ Asthma

▢ Use oxygen/respirator

▢ Seasonal allergy (pollen primarily)

▢ Horse allergy (dander primarily)

▢ Grass/hay allergy

▢ Other

▢ I am not aware of any health conditions of participants

## *Volunteer Workers*

What is the minimal volunteer to participant ratio for the indoor arena/activities space?

o 1 volunteer to 1 participant

o 2 volunteers to 1 participant

o 3 volunteers to 1 participant

o 4 volunteers to 1 participant

o Other (please specify) __________________________________________________

Approximately how many volunteers participate in a typical day at this center?

o Less than 10

o 11-25

o 26-45

o More than 46

Annually, how many volunteers participate at this center?

o Less than 25

o 26-50

o 51-100

o more than 100

If applicable, how long is the volunteer in the indoor arena/activity area on a typical day at this center?

o (Text entry)

If applicable, how long is a typical volunteer in a barn/horse housing area on a typical day at this center?

o (Text entry)

If applicable, how many hours per week does each volunteer engage in activities offered in the indoor arena/activities space?

o (Text entry)

If applicable, how many hours per week does each volunteer engage in activities offered in the barn/horse housing area?

o (Text entry)

Are you aware if the center volunteers have any of the following respiratory conditions? (select all that apply)

▢ COPD

▢ Asthma

▢ Seasonal allergy (pollen primarily)

▢ Horse allergy (dander primarily)

▢ Grass/hay allergy

▢ Other

▢ I am not aware of any health conditions of volunteers

Do volunteers receive training/education about the following respiratory conditions? (select all that apply)

▢ COPD

▢ Severe conditions (e.g. COPD, Asthma, use of artificial respirators)

▢ Mild conditions (e.g. Seasonal allergy/pollen, horse allergy/dander, grass/hay allergy, general irritation)

▢ I am not aware of any volunteer training/education in respiratory conditions

## *Horse Factors*

How many horses or horse-volunteer-participant units are in the arena space at one time?

o Less than 2 on average

o Two to four on average

o More than four on average

o Other (please specify) ________________________________________________

Are you aware if the center horses involved in activities have any of the following respiratory conditions? (select all that apply)

▢ ROA (recurrent airway obstruction)

▢ IAD (non-infectious inflammatory airway disease)

▢ Seasonal allergy

▢ No diagnosed condition, but horses will cough

▢ Other

▢ I am not aware of any health conditions of horses

Display This Question:

If Are you aware if the center horses involved in activities have any of the following respiratory c... = No diagnosed condition, but horses will cough

Which of the following do you observe to cause the most coughing in the horses?

o When in the stall/barn area

o While eating hay/grain

o During activities/interventions in the arena space

o In the pasture

Display This Question:

If Which of the following do you observe to cause the most coughing in the horses? = When in the stall/barn area

Or Which of the following do you observe to cause the most coughing in the horses? = During activities/interventions in the arena space

Which time of year do you notice the majority of the coughing in the horses?

o Winter

o Spring

o Summer

o Fall

o Year round

We are recruiting centers with at least twelve volunteers to complete specific tasks within the next year. Incentives will be provided to centers and for volunteer recruitment. To be eligible, please provide us your contact information in the open comment section below.

Please enter the center's contact information (email, contact name, website) to be considered for future studies.

________________________________________________________________

________________________________________________________________

________________________________________________________________
