# Supplementary material for: Framing Future of Work Considerations through Climate and Built Environment Assessment of Volunteer Work Practices in the United States Equine Assisted Services
Source: Int J Environ Res Public Health. 2021 Oct 2;18(19):10385. doi: 10.3390/ijerph181910385 (PMC8508050; doi:10.3390/ijerph181910385)
Supplement: Supplementary file 1 [file ijerph-18-10385-s001.zip › Tables S1 S2 S3 and S4 IJERPH 1361041 FINAL.pdf]

Table S1. Frequencies for space usage questions by climate designation.

Space Usage Questions	Cold/Very Cold	Mixed Humid	Hot Humid	Hot Dry/Mixed Dry
Which of the following best describes where you conduct the majority of your services?	n = 13	n = 12	n = 4	n = 4
Covered arena	23%	25%	75%	50%
Indoor/partially enclosed arena	0%	0%	0%	50%
Indoor/fully enclosed arena	77%	58%	25%	0%
Indoor/horse housing (stalls/barn)	0%	17%	0%	0%
($p = 0.0114$, df = 9, $X^2 = 21.297$)				
Select the activity for which you utilize your facility space the most.	n = 11	n = 12	n = 3	n = 4
Adaptive Riding/Therapeutic Riding	82%	75%	34%	75%
Hippotherapy	18%	25%	33%	0%
Recreational Riding	0%	0%	0%	25%
Competition/Boarding	0%	0%	33%	0%
($p = 0.0813$, df = 9, $X^2 = 15.366$)				
What is the minimal volunteer to participant ratio for the indoor arena/activities space?	n = 10	n = 10	n = 3	n = 3
1 volunteer to 1 participant	70%	70%	67%	100%
2 volunteers to 1 participant	20%	30%	33%	0%
3 volunteers to 1 participant	10%	0%	0%	0%
($p = 0.6823$, df = 6, $X^2 = 3.958$)				
How many horses or horse-volunteer-participant units are in the arena space at one time?	n = 12	n = 18	n = 3	n = 4
Less than 2 on average	0%	50%	33%	75%
Two to four on average	20%	50%	34%	25%
More than four on average	80%	0%	33%	0%
($p = 0.0775$, df = 6, $X^2 = 11.373$)				

Approximately how many volunteers participate in a typical day at this center?	n = 12	n = 11	n = 3	n = 4
Less than 10	33%	27%	33%	50%
11-25	25%	63%	33%	50%
26-45	42%	10%	34%	0%
(p = 0.3053, df = 6, X^2 = 7.171)				
Annually, how many volunteers participate at this center?	n = 11	n = 11	n = 3	n = 4
Less than 25	0%	27%	0%	25%
26-50	36%	0%	33%	0%
51-100	9%	9%	33%	75%
more than 100	55%	64%	34%	0%
(p = 0.0073, df = 9, X^2 = 22.552)				
If applicable, how long is the volunteer in the indoor arena/activity area on a typical day at this center?	n = 11	n = 10	n = 3	n = 3
Low (< 2 hours daily)	45%	60%	67%	67%
High (> 2 hours daily)	55%	40%	33%	33%
(p = 0.8400, df = 3, X^2 = 0.839)				
If applicable, how long is a typical volunteer in a barn/horse housing area on a typical day at this center?	n = 10	n = 10	n = 2	n = 4
Low (< 2 hours daily)	90%	50%	100%	75%
High (> 2 hours daily)	10%	50%	0%	25%
(p = 0.1431, df = 3, X^2 = 5.426)				
If applicable, how many hours per week does each volunteer engage in activities offered in the indoor arena/activities space?	n = 10	n = 10	n = 3	n = 3
Low (< 2 hours weekly)	20%	10%	33%	67%
High (> 2 hours weekly)	80%	90%	67%	33%
(p = 0.2677, df = 3, X^2 = 3.943)				

If applicable, how many hours per week does each volunteer engage in activities offered in the barn/horse housing area?	n = 10	n = 10	n = 3	n = 4
Low (< 2 hours weekly)	80%	30%	100%	75%
High (> 2 hours weekly)	20%	70%	0%	25%
(p = 0.0311, df = 3, X^2 = 28.870)				

Table S2. Frequencies for safety topic questions by climate designation.

Safety Questions	Cold/Very Cold	Mixed Humid	Hot Humid	Hot Dry/Mixed Dry
Do you have any concerns about the environment within the indoor arena/activities space?	n = 12	n = 10	n = 4	n = 4
Yes	58%	80%	100%	75%
No	42%	20%	0%	25%
(p = 0.2628, df = 3, X^2 = 3.988)				
Do your concerns fall into any of these categories?	n = 7	n = 8	n = 4	n = 3
Multiple (air movement, moisture, dust, temperature)	14%	62.5%	50%	67%
Temperature	43%	25%	0%	33%
Dust	43%	12.5%	50%	0%
(p = 0.1667, df = 6, X^2 = 9.124)				
What is the main component of the surface footing in the indoor arena/activity area?	n = 12	n = 10	n = 4	n = 4
Dirt/Clay	17%	0%	0%	0%
Sand/washed sand	42%	80%	50%	75%
Mixed (Wood Chip/Sawdust Textile/fiber)	42%	10%	25%	25%
Rubber	0%	10%	0%	0%
(p = 0.2806, df = 9, X^2 = 10.929)				
What other materials are in the footing mixture?	n = 8	n = 8	n = 3	n = 3
Dirt/Clay	25%	0%	33%	33%
Sand/washed sand	0%	0%	0%	33%
Crushed Rock/Wood Chip	12.5%	25%	0%	0%
Textile/fiber	12.5%	12.5%	0%	0%
Rubber	0%	12.5%	67%	0%
Waxed Sand	25%	0%	0%	0%
Dust suppression additive (Magnesium Chloride)	0%	50%	0%	34%
(p = 0.5423, df = 39, X^2 = 37.413)				
Who is responsible for maintaining the arena space?	n = 12	n = 10	n = 3	n = 4

Volunteers	17%	10%	0%	0%
Staff	67%	90%	100%	100%
Other (contracted labor; property owners/leased property)	16%	0%	0%	0%

(p = 0.4155, df = 6, X^2 = 6.069)

Table S3. Frequencies for organization topic questions by climate designation.

Organization Questions	Cold/Very Cold	Mixed Humid	Hot Humid	Hot Dry/Mixed Dry
Which of the following describes your Member Center affiliation?	n = 12	n = 9	n = 4	n = 4
PATH International/Premier Center Member	17%	67%	25%	50%
United States Equestrian Federation	0%	0%	25%	0%
American Hippotherapy Association	0%	11%	25%	0%
National Council for Therapeutic Recreation Certification	8%	0%	0%	0%
Multiple Affiliations (including PATH)	75%	22%	25%	50%
(p = 0.2245, df = 24, X^2 = 28.886)				
Which of the following best describes the majority of your participants who engage in your equine assisted activities or equine assisted therapies?	n = 13	n = 12	n = 4	n = 4
Children under 9 years	23%	0%	0%	25%
Children under 18 years	70%	83%	75%	50%
Adults above 18 years	8%	17%	25%	25%
(p = 0.2210, df = 9, X^2 = 11.865)				
Are you aware if the center volunteers have any of the following respiratory conditions?	n = 11	n = 9	n = 3	n = 4
Seasonal allergy (pollen primarily)	10%	55%	33%	0%
Horse allergy (dander primarily)	27%	11%	33%	25%
Grass/hay allergy	18%	0%	0%	0%
I am not aware of any health conditions of volunteers	45%	35%	33%	75%
(p = 0.2386, df = 9, X^2 = 11.572)				
Do volunteers receive training/education about the following respiratory conditions?	n = 10	n = 9	n = 2	n = 4
Severe conditions (e.g., use of artificial respirators)	0%	12%	0%	0%
Mild conditions (e.g. Seasonal, horse, grass/hay allergy)	60%	55%	50%	0%
Multiple conditions (Severe and allergy)	10%	22%	0%	50%
I am not aware of any volunteer training/education in respiratory conditions	30%	11%	50%	50%
(p = 0.3518, df = 12, X^2 = 13.241)				

Are you aware if the center horses involved in activities have any of the following respiratory conditions?	n = 11	n = 9	n = 3	n = 4
ROA (recurrent airway obstruction)	9%	0%	0%	0%
IAD (non-infectious inflammatory airway disease)	9%	0%	0%	0%
Seasonal allergy	18%	22%	0%	0%
No diagnosed condition, but horses will cough	9%	22%	67%	25%
I am not aware of any health conditions of horses	55%	56%	33%	75%
(p = 0.4529, df = 15, X^2 = 14.979)				
If horses do not have diagnosed conditions, but will cough, then which of the following do you observe to cause the most coughing in the horses?	n = 2	n = 2	n = 2	n = 1
When in the stall/barn area	50%	0%	0%	0%
During activities in the arena	50%	100%	100%	100%
(p = 0.3964, df = 3, X^2 = 2.969)				
If horse coughing is observed in the stall/barn or during activities/interventions in the arena space, which time of year do you notice the majority of the coughing in the horses?	n = 2	n = 2	n = 2	n = 1
Winter	0%	50%	0%	0%
Spring	100%	0%	50%	0%
Summer	0%	0%	50%	0%
Fall	0%	0%	0%	0%
Year round	0%	50%	0%	100%
(p = 0.1952, df = 9, X^2 = 12.333)				

Table S4. Frequencies for environmental control topic questions by climate designation.

Environmental Control Questions	Cold/Very Cold	Mixed Humid	Hot Humid	Hot Dry/Mixed Dry
Are there temperature/ventilation control methods in the indoor arena?	n = 11	n = 10	n = 4	n = 4
Fans	28%	30%	50%	75%
Heaters	27%	40%	25%	0%
None	45%	30%	25%	25%
($p = 0.5239$, df = 6, $X^2 = 2.588$)				
What type of fans are used?	n = 6	n = 6	n = 2	n = 3
Box fan	33%	17%	0%	67%
Low speed, high velocity (ceiling mounted)	50%	33%	100%	33%
Exhaust	17%	50%	0%	0%
($p = 0.2546$, df = 6, $X^2 = 3.890$)				
How many fans?	n = 5	n = 6	n = 2	n = 3
One to three	60%	100%	100%	100%
Four to six	20%	0%	0%	0%
Six to nine	20%	0%	0%	0%
($p = 0.5483$, df = 6, $X^2 = 4.965$)				
When are fans used for ventilation?	n = 6	n = 6	n = 2	n = 3
Year-round	0%	33%	0%	0%
When horses and humans are hot	33%	33%	50%	67%
Based on thermometer (set heat limits)	0%	17%	0%	0%
When it feels humid in the facility	17%	0%	0%	0%
During fly season	17%	0%	0%	0%
Multiple (temperature, dust, flies)	33%	17%	50%	33%
($p = 0.4037$, df = 24, $X^2 = 25.038$)				
